# Medical Students’ Experiences and Outcomes Using a Virtual Human Simulation to Improve Communication Skills: Mixed Methods Study

**DOI:** 10.2196/15459

**Published:** 2019-11-27

**Authors:** Timothy C Guetterman, Rae Sakakibara, Srikar Baireddy, Frederick W Kron, Mark W Scerbo, James F Cleary, Michael D Fetters

**Affiliations:** 1 Creighton University Omaha, NE United States; 2 University of Michigan Ann Arbor, MI United States; 3 Old Dominion University Norfolk, VA United States; 4 Indiana University Indianapolis, IN United States

**Keywords:** cancer, virtual reality, health communication, interprofessional relations, informatics, nonverbal communication, computer simulation, physician-nurse relations, empathy

## Abstract

**Background:**

Attending to the wide range of communication behaviors that convey empathy is an important but often underemphasized concept to reduce errors in care, improve patient satisfaction, and improve cancer patient outcomes. A virtual human (VH)–based simulation, MPathic-VR, was developed to train health care providers in empathic communication with patients and in interprofessional settings and evaluated through a randomized controlled trial.

**Objective:**

This mixed methods study aimed to investigate the differential effects of a VH-based simulation developed to train health care providers in empathic patient-provider and interprofessional communication.

**Methods:**

We employed a mixed methods intervention design, involving a comparison of 2 quantitative measures—MPathic-VR–calculated scores and the objective structured clinical exam (OSCE) scores—with qualitative reflections by medical students about their experiences. This paper is a secondary, focused analysis of intervention arm data from the larger trial. Students at 3 medical schools in the United States (n=206) received simulation to improve empathic communication skills. We conducted analysis of variance, thematic text analysis, and merging mixed methods analysis.

**Results:**

OSCE scores were significantly improved for learners in the intervention group (mean 0.806, SD 0.201) compared with the control group (mean 0.752, SD 0.198; *F*_1,414_=6.09; *P*=.01). Qualitative analysis revealed 3 major positive themes for the MPathic-VR group learners: gaining useful communication skills, learning awareness of nonverbal skills in addition to verbal skills, and feeling motivated to learn more about communication. Finally, the results of the mixed methods analysis indicated that most of the variation between high, middle, and lower performers was noted about nonverbal behaviors. Medium and high OSCE scorers most often commented on the importance of nonverbal communication. Themes of motivation to learn about communication were only present in middle and high scorers.

**Conclusions:**

VHs are a promising strategy for improving empathic communication in health care. Higher performers seemed most engaged to learn, particularly nonverbal skills.

## Introduction

### Background

Communication is critical to health encounters. Poor communication in health care has been linked to adverse consequences, medical errors, and decreased satisfaction [1-6]. Both patient-provider and provider-provider communication skills contribute to outcomes. Communication is foundational to many aspects of the health encounter from imparting information and diagnoses to offering explanations for medical decision making. The quality of those explanations (ie, *causability*) relies on high-quality communication [7]. In addition to imparting and gathering information, the manner in which providers communicate is equally important and often underemphasized. To address this critical need, MPathic-VR is an intervention that leverages virtual human (VH) informatics technology to target training empathic communication skills. Empathy is a set of constructs that relate to the “response of one individual to the experiences of another” [8]. In contrast, sympathy is typically a reference to feeling of compassion for others. Constructs of empathy include the following: (1) antecedents—individuals and situations, (2) processes—mechanisms to produce an empathic outcome, (3) intrapersonal empathic outcomes—cognitive and emotional responses within the observer, and (4) interpersonal empathic outcomes—behavioral responses of the observer directed toward the target [8]. These definitions assume 2 or more individuals, an observer (eg, a provider) who is in a situation and is responding empathically and a target individual (eg, a patient) with whom the observer interacts.

Teaching empathy includes both cognitive and emotional domains [9,10]. “Empathy is the foundation of patient care” [10] that is cultivated through actively listening to patients. Despite its foundational importance, training in empathy receives relatively little attention and even erodes during medical school [11].

Communicating with empathy requires attention to both verbal and nonverbal communication, such as microencouragers, proximity, eye contact, nodding, and appropriate use of smiles. Interventions with providers tend to give skills training in a variety of formats, such as videos, courses, or workshops [12,13]. Computer-based conversational agents have been developed to address cognitive tasks [14] such as verbal communication [15,16] and reasoning for diagnosis and therapy. A useful conversational agent is VHs, which have human appearance and the ability to interact, responding to humans and engaging in communication behaviors as in a typical conversation [17].

The body of VH literature related to health communication training is relatively small. Yet, research on VH to enhance health communication shows promise in training medical students and nurses [15,18-21]. VHs offer a unique advantage by providing an authentic yet low-risk simulated environment to learn with the appropriate level of challenge [22]. Users perceive VH interactions as real [23] social situations, and they perceive that authenticity can enhance learning and engagement [22,24,25]. The underlying informatics system can direct the learner through an adaptive path through a scenario based on responses. These systems collect data (eg, verbal responses) as the learner interacts with the VH, process the interaction, and provide real-time automated feedback to the learner. After receiving formative feedback, the learner can immediately reflect and repeat the simulation experience, which changes depending on the learner’s actions. Reflection and deliberate practice are crucial to developing communication skills [26-29].

In contrast to human standardized patients, by leveraging informatics technology, VHs present information in a consistent manner, eliminating the variability of repeated human performances without becoming fatigued. As an informatics-based system, VHs could interpret nuances of nonverbal behavior and facial expression dynamically and then present its formative feedback to learners in the after-action review, eliminating the need for labor-intensive video analysis and conversation analysis.

### Virtual Human Communication Simulation

In a parent study, a multisite single-blinded mixed methods randomized controlled trial [30], medical students were randomly assigned to 1 of the 2 conditions: an MPathic-VR intervention or a computer-based learning control module [30]. MPathic-VR is an informatics-based technology that engages learners in a conversation with VH characters to provide training in both verbal and nonverbal communication. Using active learning strategies, the simulation is designed for learners to have authentic, challenging conversations with VHs. The simulation uses intelligent VHs that detect body motion, facial expressions, and speech. The system consists of a computer with a widescreen monitor, a microphone to recognize speech, and a Microsoft Kinect sensor to detect predefined nonverbal communication behaviors (ie, smiles, nodding, body leaning, and eyebrow raises). MPathic-VR trains the learner in nonverbal behaviors, which the user must demonstrate to the system as picked up by a sensor before continuing.

The intervention involved interactive modules with 3 VHs: a young Latina woman who developed leukemia, her mother, and a nurse providing care for the woman. The learner participated in 2 scenarios—intercultural and interprofessional—that required expressing empathy and demonstrating nonverbal listening skills. At points needing communication from the learner, each scenario paused, and the system presented 3 possible responses. The system recognized the response the learner provided to the VH, which led to a commensurate path in the scenario (eg, a poor response would escalate the situation). After completing each full scenario, the system provided automated feedback, and the learner repeated the scenario. Medical students randomized to the intervention were assigned an MPathic score based on their performance.

After completing the intervention or control, students wrote qualitative reflections about their experience. Moreover, 1 to 2 weeks later, all students completed an objective structured clinical examination (OSCE). As previously reported, the primary outcome of the trial showed that MPathic-VR has a positive, statistically significant effect on medical student’s proficiency in disclosing a new cancer diagnosis (intercultural scenario) and practicing conflict resolution in an interprofessional scenario with an oncology nurse [31].

As the trial demonstrated effectiveness of MPathic-VR intervention, we turned our focus to understanding the mechanisms and differential experiences with the MPathic-VR intervention. The aim of this study was to focus on only students exposed to the MPathic-VR simulation in the intervention arm and investigate differential effects of the simulation. Our primary aim was to answer the question: *How do medical student reflections about their experiences compare between low, medium, and high performers on the primary outcome measures of communication performance in the simulation and on the OSCE?*

## Methods

### Design

This study employed a convergent mixed methods design involving integrating 2 quantitative measures (MPathic-VR–calculated scores and the OSCE scores) with qualitative reflections by learners about their experiences. Examining qualitative reflections helped to elucidate educational mechanisms for high, medium, and low OSCE scoring participants. The full randomized controlled trial results are reported elsewhere [31]. The University of Michigan Institutional Review Board (HUM00134766) determined that the study was exempt under the educational research category and waived documentation of consent for students.

### Setting

The intervention took place at Eastern Virginia University Medical School, the University of Michigan Medical School, and the University of Virginia School of Medicine.

### Participants

Second-year medical students from the 3 medical schools were recruited and randomly assigned to the control (n=211) or MPathic condition (n=210), the MPathic group is the focus of this study. We excluded 4 individuals because of missing qualitative data, leaving 206 students for this analysis.

### Qualitative Data Collection

We employed an ethnographically driven approach for the qualitative component of the study as we sought to understand the nature of their experiences with the system. The students in each condition completed a reflective essay on their experience. Students were randomized to 1 of the 5 reflective questions about (1) human interactions, (2) understanding nonverbal communication, (3) most important things learned, (4) how to improve the simulation, and (5) functional aspects.

### Qualitative Data Analysis

We imported all reflective responses into MAXQDA qualitative software (VERBI) to manage data for further analysis. We then followed a thematic qualitative text analysis [32] process. The first step was to review the entire database while memoing potential themes and connections among the data [33]. Next, we began coding the responses by assigning descriptive labels to segments of text and generated an initial codebook. A total of 3 individuals coded (RS, SB, and TG) data and met to review codes and reach agreement through a consensus process [34]. We paused to review the codes, discussed discrepancies to create a common code definition, eliminated redundant codes, and refined descriptions for each code. Using this codebook, we proceeded with systematically coding the remainder of the database but also allowed additional codes to emerge as needed. Finally, we grouped similar codes into themes and considered the relationship among themes.

### Quantitative Data Collection

MPathic-VR scores (continuous data) were collected that reflect the path through the system and responses for each participant. For each exchange with the VH, the system recorded a point value (3-point scale of optimal to worst with lower scores reflecting better performance) and summed points for an overall MPathic-VR score for each scenario.

Students’ advanced communication skills were assessed through an OSCE around a novel scenario, although all students knew they would be tested. Standardized patient instructors evaluated each student’s performance on a 5-point scale across 4 domains: open or defensive, collaborative or competitive, nonverbal communication, and an awareness of others.

### Quantitative Data Analysis

One score was missing for the interprofessional scenario. Using an analysis of variance, we compared OSCE scores for the MPathic-VR and control condition. In addition, we examined changes in the MPathic score upon first run through the scenario and the repeat run after receiving feedback. Statistical significance was determined by a 95% CI.

### Mixed Methods Integration

The value of mixed methods research lies in meaningful integration of qualitative and quantitative components. We employed merging integration, which consisted of comparing qualitative findings with respect to OSCE and MPathic scores and determining whether the qualitative findings confirmed, disconfirmed, or expanded our understanding of the scores [35]. Data were not normally distributed, so we converted each OSCE and MPathic score into a 3-level categorical variable (high, medium, and low). On the basis of tertiles, OSCE scores of less than 0.57 were categorized as low, 0.57 to 0.95 as medium, and greater than 0.95 as high. MPathic scores from the second run-through for each scenario were converted into categorical variables. Keeping in mind that low MPathic scores reflect better performance, for the intercultural scenario, scores of less than 3 were categorized as high, 3 to 7 as medium, and greater than 7 as low. In the interprofessional scenario, scores of less than 3 were categorized as high, 3 to 6 as medium, and greater than 6 as low. We used joint display analysis by arraying qualitative themes by OSCE and MPathic scores to understand how the experience differed among participants at these OSCE and MPathic score levels.

## Results

### Qualitative Results

Our analysis identified 3 major positive themes for the MPathic-VR group learners: gaining useful communication skills, learning awareness of nonverbal skills in addition to verbal skills, and feeling motivated to learn more about communication. A subset of participants expressed some reservations about this initial version of the system encapsulated by subthemes of potential improvements: uncertainty about timing/suitability of training with the VHs in the system versus actual humans, questions about the *repetition*, and a minority disinterested in any communication training.

#### Gaining Useful Communication Skills

Regarding verbal communication, learners reported gaining strategies for effectively interacting with patients and other health care providers in real clinical settings. Frequently mentioned strategies included asking open-ended questions, validating or acknowledging the conversation partner’s feelings with reflective language, and remembering the importance of a simple apology. A few reported that the time built in to consider their verbal response and the immediate feedback helped them carefully think about their word choices before speaking. Specifically, learners reported being more mindful of selecting words that display cultural humility (avoiding assumptions), using inclusive language (*we* or *together* vs *I*), and avoiding *yes but* sentences (nonconfrontational phrasing).

#### Learning Awareness of Nonverbal Skills

Learners reported becoming more cognizant of their facial expressions in conversation, especially eyebrow movement, nodding, and smiling. Some learned about their own unfavorable behavior that they were not previously aware of, such as twitching and fidgeting. Beyond personal awareness, learners mentioned that they became aware of the importance of nonverbal communication in establishing rapport and conveying interest, acknowledgment, and empathy.

#### Feeling Motivated to Learn More About Communication

Learners noted that they would benefit from more training like MPathic-VR and expressed interest in interacting with the system through different scenarios. Learners were interested in additional scenarios that involve noncompliant or angry patients, determining a care plan, and delivering difficult diagnoses.

#### Optimizing the System

A subset of participants expressed either some reservations about the initial version of the system or disinterest in communication training. As the themes represented a small proportion, we labeled them as subthemes. One subtheme is the uncertainty about VH rather than humans for training. A few learners commented on the mechanics of talking with a VH owing to the lack of variability in conversation because of the multiple-choice responses and the interruption in conversation and eye contact to read the prompts on screen. Another subtheme was that the training was *too repetitive* by having individuals practice talking and repeat each scenario after receiving feedback. These individuals reported losing interest in the exercise when having to complete the scenario for the second time.

Disinterest in the training included both feeling “I already know how to communicate” and doubting the importance of nonverbal behaviors. A few learners felt as if the lessons provided were “common sense*”* and did not expand on their current knowledge, reflecting a broader lack of interest in communication skill building. Some learners mentioned that the system’s prompt to use nonverbal behavior made the interaction “phony.”

### Quantitative Results

The MPathic score for the group randomly assigned to the training intervention is based on responses through each scenario, and it indicated a statistically significant improvement from the initial to the repeat scenario after feedback. Scores improved (a lower score is better) from the first (mean 11.67, SD 6.26) to the second time through the intercultural scenario (mean 5.89, SD 5.12; *F*_1,207_=166.14; *P*<.001), and scores improved for the interprofessional scenario from the first (mean 7.59, SD 3.96) to the second time (mean 4.62, SD 2.54; *F*_1,207_=104.64; *P*<.001). The global OSCE score was better for the MPathic-VR condition than the control condition, as reported in detail previously (*F*_1,414_=6.09; *P*=.01) [31]. The nonverbal subdomain was also significantly higher for the MPathic-VR condition (*F*_1,414_=13.70; *P*<.001) [31].

### Integrated Mixed Methods Results

#### Primary Aim: Objective Structured Clinical Examination Score and Qualitative Comments

The distribution of medical students according to lowest, medium, and highest scoring participants on the OSCE was 10.7% (22/206), 66.0% (136/206), and 23.3% (48/206), respectively. We examined whether any patterns were present among qualitative themes by the lowest, medium, and highest scoring participants on the OSCE, as reported in Table 1. Although we did not find differences for all themes, several noteworthy patterns emerged.

Learners in all 3 groups commented on learning useful verbal and nonverbal communication skills, although low OSCE scorers had fewer comments compared with medium and high OSCE scorers. Most of the variation between groups was noted in comments about nonverbal behaviors. Interestingly, several medium and high OSCE scorers specifically commented on learning how to use nonverbal communication where appropriate, whereas only 1 low scorer mentioned nonverbal aspects. Examples included using body language to build rapport and replacing verbal responses with nonverbal cues to avoid interrupting the flow of the conversation. Many leaners also commented on being mindful when nodding and smiling, noting that these behaviors can be misinterpreted in certain situations as insensitive or arrogant.

**Table 1:** A joint display of qualitative themes by quantitative performance level on an objective structured clinical examination.

Themes	Objective structured clinical examination advanced communication assessment		
	Low (<0.55)	Medium (0.54-0.98)	High (>0.98)
Useful communication skills	N/A^a^	“Effective communication both verbal and nonverbal will be essential in getting the best care for patients.”	“I thought that I was given helpful strategies for interacting with patients such as asking open-ended questions, validating feelings, and types of nonverbal cues to use.”
Remembering nonverbal skills	“Smiling and nodding is also important”	“Non-verbal cues can be very helpful. There are good times to nod and also times when it is not appropriate.” and “In emotionally charged situations, I realize that using non-verbal communication is very important.”	“Helped teach how to read facial expressions from people such as when the nurse was upset.”
Motivated to learn more	N/A	“I would definitely benefit from more training such as this. I found myself hoping that there would be another simulation or two.”	“It would be interesting to go through other scenarios, and to see if this actually has a positive effect on my future interactions with patients.”
Prefer humans	“Hard to engage in non-verbal communication when you know you are just talking at a computer.”	“I think that training for communication with patients is better done with live patients.”	“Your true response can only come from human to human interaction…program is much stronger at allowing a person to think about their verbal responses.”

^a^N/A: not applicable.

Medium and high OSCE scorers also reported gaining nonverbal skills in managing emotionally charged and complex situations (eg, family politics in health care), which was not discussed by low scorers. High scorers also noted the importance of mirroring their conversation partner’s facial expressions to help diffuse tense situations. Low scorers did not provide comments that indicated awareness of their conversation partner’s nonverbal behaviors.

Expressions of motivation to learn about communication were made only by medium and high OSCE scorers. However, reservations about training arose from some of these learners as well. Notably, several medium OSCE scorers and 1 high scorer questioned the value of the training relative to their time. They explained that they were unable to fully immerse themselves in the exercise because they were distracted by external factors such as exams. Comments about already knowing how to communicate were also only made by medium and high OSCE scorers.

Comments from some members of all 3 groups questioned the role of VHs for communication training versus humans. We found no difference among the 3 performance groups in discussing realism of the VH training experience.

#### Secondary Aim: MPathic Scores and Qualitative Comments

The distribution of medical students according to lowest, medium, and high scores on the intercultural MPathic simulation participants was 18.4% (38/206), 60.2% (124/206), and 21.4% (44/206), respectively. The distribution of medical students according to lowest, medium, and high scores on the interprofessional MPathic simulation participants was 18.9%) (39/206), 64.6% (133/206), and 16.0% (33/206), respectively. One score was missing for the interprofessional scenario. We investigated whether any patterns were present among qualitative themes by low, medium, and high scoring participants in MPathic scores for both intercultural and interprofessional scenarios. In both scenarios, patterns were similar to those that emerged when comparing low, medium, and high OSCE scorers against their qualitative experiences. High, medium, and low group membership overlapped for both the OSCE and MPathic scores (eg, high OSCE and MPathic scores). In the intercultural scenario, 12 learners were in the high scoring group for both OSCE and MPathic scores, 81 were in the middle scoring group, and 6 were in the low scoring group. In the interprofessional scenario, 6 were in the high scoring group, 87 were in the middle scoring group, and 4 were in the low scoring group for both OSCE and MPathic scores.

In both scenarios, learners across all groups acknowledged the use of appropriate nonverbal behaviors. Learners in all groups also mentioned that they learned how to use nonverbal behavior to help manage tense situations. However, when comparing low, medium, and high OSCE scores against qualitative themes, these were mentioned mostly by high OSCE performers and only 1 low OSCE performer.

When comparing the qualitative comments made by low, medium, and high scorers between the 2 MPathic scenarios, similar patterns emerged. For the intercultural scenario, comments indicating a desire to learn more about communication were made across all groups, although there were more mentions from high performers compared with low performers. In contrast, in the interprofessional scenario, low and middle performers had more of a desire to learn about communication compared with high performers.

#### Improvement in MPathic Scores and Qualitative Comments

Finally, we compared comments by whether learners improved their MPathic scores. The distribution of pre-post change for the intercultural scenario included improved scores (85.4%, 176/206), no change (7.3% (15/206), and worse performance (7.3%, 15/206). For the interprofessional simulation, the distribution was 77.7% (160/206), 10.2% (21/206), and 11.7% (24/206), respectively. In both scenarios, learners who did not improve or those who did worse on the second run-through made comments about engagement in the training that related to a lack of interest. These learners indicated that they would rather use the MPathic training time to study for other courses. In contrast, those who improved their scores were more likely to mention engagement issues that related to training procedures, such as being unaccustomed to gesturing to a computer screen. In both scenarios, learners who did worse on the second run-through did not want to learn more or practice more.

## Discussion

### Principal Findings

Several qualitative differences in themes were apparent when comparing high, middle, and lower performing individuals based on posttraining OSCE communications performance scores. The higher scoring individuals noted the importance of learning about communication and communicating appropriately more than lower scoring individuals. Compared with lower performers, higher performers emphasized the importance of verbal and nonverbal communication skills when interacting in health settings. The pattern was especially notable in their reflection of nonverbal communication. Overall, the integrated results suggest that higher performing individuals seemed to understand and perhaps have stronger buy-in as to the importance of health communication and motivation to learn further.

Regarding our secondary aims of examining how learners compared based on their overall performance during the simulation, and on the first and second runs, learners who did not improve or performed worse on the second run-through in both scenarios expressed more dissatisfaction with the learning experience. These learners showed no interest in wanting to learn more about communication or practicing with the MPathic training system or perhaps did not understand the importance of communication training. This pattern was not as blatant when comparing qualitative themes by low, medium, and high performers in OSCE and MPathic scores. These learners may simply not be good candidates for the training because of personal factors, such as competing priorities. However, in an actual implementation, MPathic-VR could be available on demand at any time needed to fit the learner’s schedule. In addition, these individuals may have had low motivation to participate, which had a negative effect on learning outcomes [36], for example, a subset doubted the importance of nonverbal skills. A potential strategy is to increase upfront information and explain that nonverbal expression transmits a larger portion of information than words. Ironically, such students may benefit the most from communications training.

### Comparison With Prior Work

Recent research has examined the value of virtual patients, broadly, applied to improve medical decision making, such as creating accurate virtual patient cases derived from electronic health records to train decision making [37]. Our work further adds to the literature about VHs, focusing specifically on communication training, critical to decision making and the entire health care encounter. Our overall posttraining measures indicated a favorable benefit of VH training, similar to results of other VH interventions to improve verbal communication skills, such as VHs to enhance providers’ ability to impart knowledge about appropriate antibiotic use and improve views of shared decision making [15]. Our study, however, also investigated effects on nonverbal communication skills and found promising outcomes. Although nonverbal skills were assessed, learner feedback is not yet automated, as in Liu et al’s Web-based system used to provide automated nonverbal feedback [38].

The unique contributions of this study arise from using a mixed methods approach and focusing our attention on only students exposed to the MPathic-VR simulation to understand the mechanisms by which learners interacted with the intervention. In our investigation, merging of qualitative and quantitative databases revealed confirmation of findings related to intervention differential effects and mechanisms of action. Furthermore, visualization of these findings through the use of a joint display facilitated our interpretation of findings (Table 1). One mechanism related to the intervention was motivation. We found less favorable OSCE and MPathic scores among learners who expressed a lack of interest in the training in their written reflection. Thus, motivational issues seemed to be an important human factor related to engagement with the informatics technology. This result has implications in either priming health professionals before participating or selecting who is most likely to benefit. Another mechanism is awareness of nonverbal communication. Learners who explained that they gained skills in the appropriate use of nonverbal communication to manage emotionally charged situations tended to achieve better intervention outcomes (ie, favorable OSCE). Confirmation of findings with both quantitative and qualitative methods lends credibility to our understanding of these MPathic intervention mechanisms [35]. Furthermore, results suggest that humans will engage in nonverbal communication when interacting with an informatics-driven intelligent VH. When appropriate, nonverbal communication might be considered among the human factors principles in conducting research on interaction with informatics technology.

### Study Limitations

Our study was limited to medical students, and future research is needed to apply the simulation to practicing providers and other health professionals. In addition, nonverbal communication was not the focus of data collection despite arising as a primary finding. Training nonverbal communication skills in VH simulation merits further investigation. In addition, the nonverbal behaviors specifically selected for this simulation—nodding, smiling, and eyebrow raises—are a few of the many possible nonverbal and verbal behaviors associated with empathy expression [39]. Coding schemes that offer methods for meticulously analyzing nonverbal behaviors and their associated emotions exist and can be applied to the Kinect sensor data collected in the trial [40,41].

### Conclusions

Future research should address how to motivate and engage learners. On one hand, the MPathic system has made an innovative leap forward by demonstrating the effect of the system to train communication skills that transfer into a realistic communication scenario. The results raise research questions about the need to incorporate instructional design principles that will help motivate students skeptical about improving their health care communication [42]. Although the results indicated that higher performers had stronger beliefs about the importance of good communication in health care, it is unclear whether these differences were present before participating in the intervention. Future research may benefit from additional pretest measures, such as a written reflection about communication and empathy assessments, such as the Jefferson Scale [43].

## Abbreviations

OSCE: objective structured clinical examination
VH: virtual human
